# Primary Myeloid Sarcoma of Ovary: A Case Report and Review of the Literature

**DOI:** 10.5146/tjpath.2020.01517

**Published:** 2021-09-15

**Authors:** Sevda Akyol, Fatma Oz Atalay

**Affiliations:** Department of Pathology, Hakkari Yuksekova State Hospital, Hakkari, Turkey; Department of Bursa Uludag University Medicine Faculty, Bursa, Turkey

**Keywords:** Myeloid sarcoma, Ovary, Female genital neoplasms

## Abstract

Myeloid sarcoma (granulocytic sarcoma or chloroma) is a tumor formed by myeloid precursor cells in any localization other than the bone marrow. It can occur without underlying acute myeloid leukemia (AML) or other myeloid neoplasms. Herein, we present a forty-two-year-old female patient who underwent surgery because of a left adnexal mass. Microscopic examination of the specimen revealed cord-like arrangement of the tumor cells with a diffuse growth of small blue cells effacing the ovarian stroma. Adult granulosa cell tumor was in the differential given the scanty cytoplasm of the tumor and in fact was the diagnosis of the referring institution. Further microscopic evaluation with immunohistochemical analysis at our institution revised the diagnosis to myeloid sarcoma. Myeloid sarcoma is a difficult tumor to diagnose due to its rarity, especially in the absence of a history of leukemia, and correct tissue diagnosis is essential for its treatment.

## INTRODUCTION

Myeloid sarcoma (granulocytic sarcoma, chloroma) is a tumor formed by myeloid precursor cells in any localization other than the bone marrow (1). It was first described in the literature by Burns in 1811 (2). When the tumor is exposed to oxygen, it turns green. Due to this color of the tumor, it was named chloroma derived from the Greek word chlorosis (green) (3).

Myeloid sarcoma can occur without underlying acute myeloid leukemia (AML) or other myeloid neoplasms. It can be seen anywhere on the body but skin, lymph node, gastrointestinal tract, bone, and testicle placement is common (4,5). Only a few cases of myeloid sarcoma localized in the female genital system (cervix, vagina, vulva, ovary, and endometrium) have been reported in the literature (6–8). In this case report, we presented a case of primary ovarian myeloid sarcoma without an apparent, previously diagnosed hematologic malignancy.

## CASE REPORT

A forty-two-year-old female patient was admitted to another center with pain in the abdomen. The patient was followed up with psoriasis and had no other known systemic diseases. Upon detecting a mass in the left adnexal area in the radiological images, total abdominal hysterectomy and bilateral salpingo-oophorectomy was performed.

**Figure 1:**
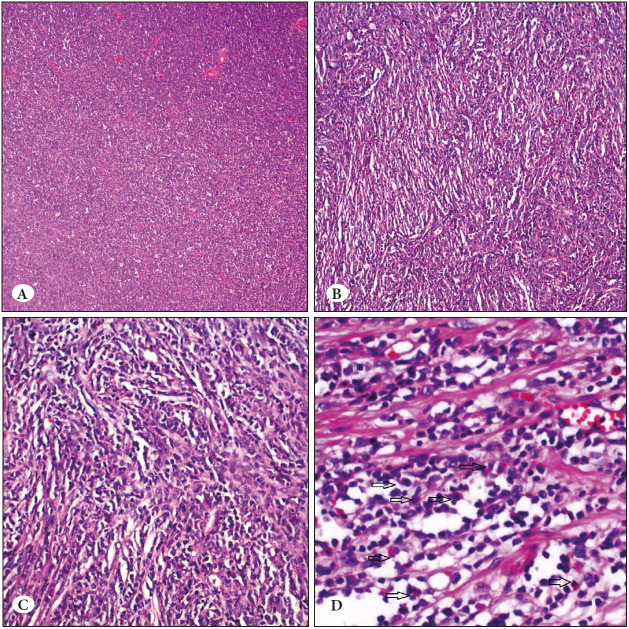
**A)** Areas with sheet-like infiltration by small-blue tumor cells (H&E; x40). **B-C)** Tumor cells consisting of cord structures separated by fine fibrous septa (H&E; x100&200). **D)** The presence of eosinophilic myelocytes among tumor cells is remarkable (H&E; x200).

Grossly, the tumor was located in the left adnexa and was 9 cm in the greatest dimension. The cut surface was solid with areas of necrosis. The microscopic examination showed a diffuse infiltrative growth pattern with tumor cells focally forming cord-like structures. The tumor was poorly differentiated with 50 mitoses per 10 high power fields (HPF). The widespread necrosis seen by gross examination was also appreciated by microscopic evaluation. The tumor cells were mostly uniform with little cytoplasm and hyperchromatic nuclei, and focally cells with eosinophilic cytoplasm with vesicular nuclei and conspicuous nucleoli and ill-defined cytoplasmic borders were present (Figure 1).

**Figure 2:**
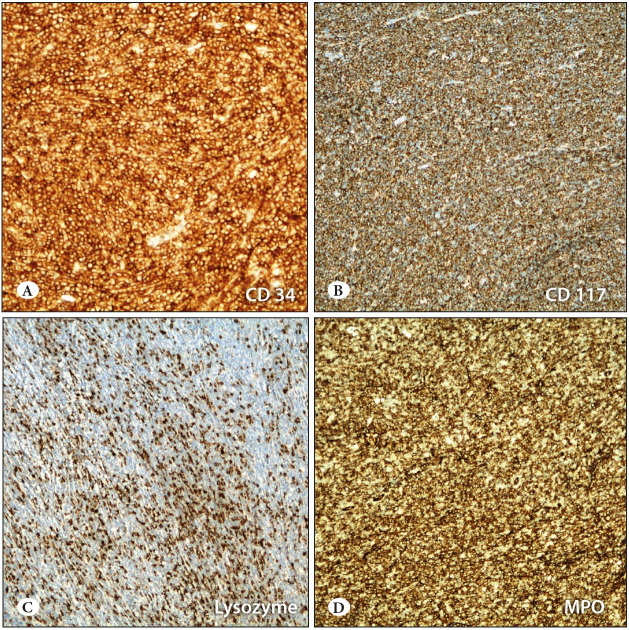
Strong positivity to immunohistochemical staining with **A)** CD34 (IHC; x100). **B)** CD117 (IHC; x100). **C)** Lysozyme (IHC; x100). **D)** MPO (IHC; x100).

The tumor was initially thought to be a granulosa cell tumor and referred to our institution for further work-up. Given the poorly-differentiated nature of the tumor, the differential was broad and included lymphoma, metastatic breast carcinoma, poorly differentiated carcinoma (metastatic or primary), melanoma, or an unusual presentation of a malignant mesenchymal tumor. Tumor cells showed widespread positive reactivity with LCA, CD117, MPO, CD43, lysozyme, CD34, and CD68 in the immunohistochemical stainings (Figure 2). No immune reactivity was observed with PanCK, vimentin, CD3, CD20, EMA, SF-1, WT-1, inhibin, or calretinin. As a result of the morphological and immunohistochemical studies, the case was reported as myeloid sarcoma.

## DISCUSSION

Ovarian tumors are classified histopathologically as epithelial tumors, mesenchymal tumors, mixed epithelial and mesenchymal tumors, sex cord-stromal tumors, mixed sex cord-stromal tumors, germ cell tumors, germ cell-sex cord-stromal tumors, mesothelial tumors, soft tissue tumors, lymphoid and myeloid tumors, miscellaneous tumors, tumor-like lesions and secondary tumors (9). Ninety percent of malignant ovarian tumors are of epithelial origin (10). However, when the literature was examined, it was observed that there were less than 50 cases of myeloid neoplasms of the ovary.

The first primary ovarian myeloid sarcoma was published in 1959 (11). Twenty-five cases have been reported as case reports (12–33). Two relevant series comprising 19 cases were published (34,35). The patient ages in the case reports ranged from 3 months to 58 years. The mean age of the patients was 32.1 years (18–33). Most of the patients were in the reproductive age. The age of our patient was 42 years. The patients presented with complaints of abdominal pain (8/15), mass (2/15), cough (1/15), and vaginal bleeding (1/15), and the pathology was detected in 3 patients (3/15) during a routine check (20–33). The tumor was located in the left ovary in 5 cases, the right ovary in 8 cases, and bilaterally in 3 cases (15,19,22,23). In our case, it was located in the left ovary. The tumor diameter ranged from microscopic size to 25 cm (25,28). The size of the tumor in our case was 9x8.5 cm.

Ovarian myeloid sarcoma may occur before AML, simultaneously with AML, as a result of the recurrence of AML, or as an isolated tumor mass. It may also be associated with myelodysplastic syndrome or myeloproliferative neoplasm (34). Three of the cases were found to be isolated myeloid sarcoma (15,25,33). After myeloid sarcoma diagnosis, AML developed in 8 cases (16,21–23,26,30–32). Bone marrow involvement was seen simultaneously with myeloid sarcoma in 2 cases (20,28). Before the mass developed in the ovary, six patients had AML and 1 patient had chronic myeloid leukemia (14,17,19,24,29). The time of the systemic disease with medullary involvement following the initial myeloid sarcoma diagnosis ranged from just after surgery to 18 months afterwards (26,31).

Diagnosing myeloid sarcoma in the absence of a history of leukemia can be challenging. It is reported that only 44% of myeloid sarcoma cases can be diagnosed correctly at the first examination (36). In our case, it was misdiagnosed as a granulosa cell tumor at another center at the first examination. Granulosa cell tumor may be considered first when a blue cell tumor with scant cytoplasm is seen in the ovary. However, the cells are angulated nuclei and some have grooves similar to coffee beans in granulosa cell tumor. The tumor may exhibit various architectural patterns such as trabecular, diffuse and microfollicular. Even if a high-grade transformation is encountered in some tumors, there are still low-grade areas containing typical morphological features separated by an abrupt transition. Abundant eosinophilic cytoplasm and prominent nuclear atypia are observed in tumor cells in the high-grade areas. The nuclei can have a bizarre multinucleated form (37).

Myeloid sarcomas are less differentiated and the differential diagnosis spectrum is therefore quite wide. Diffuse large B cell lymphoma, Burkitt’s lymphoma, undifferentiated carcinoma, metastatic lobular carcinoma, small cell carcinoma of the hypercalcemic type, granulosa cell tumor, and dysgerminoma can be included in the differential diagnosis. Immunohistochemical studies can be very helpful during the differential diagnosis. LCA positive staining and CD3 and CD20 negative staining eliminate the diagnosis of lymphoma. Negative staining with panCK excludes the diagnosis of metastatic carcinoma and undifferentiated carcinoma. Other diagnoses are excluded with CD56, synaptophysin, calretinin, inhibin, SF-1, and PLAP negativity. CD34, MPO, CD117, CD68, and lysozyme positivity also support the diagnosis of myeloid sarcoma.

In conclusion, we presented a case of myeloid sarcoma in the ovary, which has been published in the literature only in very small numbers. It is important to know the patient’s history and consider this tumor entity to make a correct diagnosis.

## Conflict of INTEREST

The authors declare no conflict of interest.
